# COllaborative Neuropathology NEtwork Characterizing ouTcomes of TBI (CONNECT-TBI)

**DOI:** 10.1186/s40478-021-01122-9

**Published:** 2021-03-01

**Authors:** Douglas H. Smith, Jean-Pierre Dollé, Kamar E. Ameen-Ali, Abigail Bretzin, Etty Cortes, John F. Crary, Kristen Dams-O’Connor, Ramon Diaz-Arrastia, Brian L. Edlow, Rebecca Folkerth, Lili-Naz Hazrati, Sidney R. Hinds, Diego Iacono, Victoria E. Johnson, C. Dirk Keene, Julia Kofler, Gabor G. Kovacs, Edward B. Lee, Geoffrey Manley, David Meaney, Thomas Montine, David O. Okonkwo, Daniel P. Perl, John Q. Trojanowski, Douglas J. Wiebe, Kristine Yaffe, Thomas McCabe, William Stewart

**Affiliations:** 1 Center for Brain Injury and Repair, Department of Neurosurgery, Perelman School of Medicine, University of Pennsylvania, Philadelphia, PA USA; 2 Institute of Neuroscience and Psychology, University of Glasgow, Queen Elizabeth University Hospital, Glasgow, UK; 3 Department of Biostatistics, Epidemiology and Informatics, Perelman School of Medicine, University of Pennsylvania, Philadelphia, PA USA; 4 Department of Pathology, Icahn School of Medicine At Mount Sinai, New York, NY USA; 5 Neuropathology Brain Bank and Research Core, Ronald M. Loeb Center for Alzheimer’s Disease, Departments of Pathology and Neuroscience, Icahn School of Medicine, New York, NY USA; 6 Department of Pathology, Fishberg Department of Neuroscience, Friedman Brain Institute, Icahn School of Medicine At Mount Sinai, New York, NY USA; 7 Department of Rehabilitation Medicine, Icahn School of Medicine At Mount Sinai, New York, NY USA; 8 Department of Neurology, Icahn School of Medicine At Mount Sinai, New York, NY USA; 9 Department of Neurology, University of Pennsylvania Perelman School of Medicine, Philadelphia, USA; 10 Center for Neurotechnology and Neurorecovery, Department of Neurology, Massachusetts General Hospital, 175 Cambridge Street – Suite 300, Boston, MA USA; 11 Athinoula A. Martinos Center for Biomedical Imaging, Department of Radiology, Massachusetts General Hospital and Harvard Medical School, Charlestown, MA USA; 12 Department of Forensic Medicine, New York University School of Medicine, New York, NY USA; 13 Department of Laboratory Medicine and Pathobiology, University of Toronto, Toronto, ON Canada; 14 Department of Pathology, The Hospital for Sick Children, Toronto, ON Canada; 15 Canadian Concussion Center, Toronto, ON Canada; 16 Brain Tissue Repository and Neuropathology Core, Center for Neuroscience and Regenerative Medicine (CNRM), Uniformed Services University (USU), Bethesda, MD USA; 17 Chronic Effects of NeuroTrauma Consortium (CENC), Fort Detrick, MD USA; 18 The Henry M. Jackson Foundation for the Advancement of Military Medicine (HJF), Bethesda, MD USA; 19 Department of Laboratory Medicine and Pathology, University of Washington, Seattle, WA USA; 20 Department of Pathology, University of Pittsburgh School of Medicine, Pittsburgh, PA USA; 21 Tanz Centre for Research in Neurodegenerative Disease (CRND) and Department of Laboratory Medicine and Pathobiology, University of Toronto, Krembil Discovery Tower, 60 Leonard Ave, Toronto, ON Canada; 22 Laboratory Medicine Program and Krembil Brain Institute, University Health Network, Toronto, ON Canada; 23 Department of Pathology and Laboratory Medicine, Perelman School of Medicine, University of Pennsylvania, Philadelphia, PA USA; 24 Department of Neurological Surgery, University of California San Francisco, 505 Parnassus Ave, Rm M779, San Francisco, CA USA; 25 Brain and Spinal Injury Center, Zuckerberg San Francisco General Hospital, 1001 Potrero Ave, Bldg. 1, Rm 101, San Francisco, CA USA; 26 Department of Bioengineering, University of Pennsylvania, Philadelphia, PA USA; 27 Department of Neurosurgery, University of Pennsylvania, Philadelphia, PA USA; 28 Department of Pathology, School of Medicine, Stanford University, Palo Alto, CA USA; 29 Department of Neurological Surgery, University of Pittsburgh, Pittsburgh, PA USA; 30 DoD/USU Brain Tissue Repository and Neuropathology Core, F. Edward Hébert School of Medicine, Uniformed Services University (USU), Bethesda, MD USA; 31 Department of Pathology and Laboratory Medicine, Center for Neurodegenerative Disease Research, Perelman School of Medicine, University of Pennsylvania, Philadelphia, PA USA; 32 Department of Neurology, Epidemiology and Biostatistics, University of California San Francisco, San Francisco, CA USA; 33 School of Medicine, Dentistry and Nursing, University of Glasgow, Glasgow, UK; 34 Department of Neuropathology, Queen Elizabeth University Hospital, 1345 Govan Rd, Glasgow, G51 4TF Queen UK

**Keywords:** Traumatic brain injury, Chronic traumatic encephalopathy, Neurodegenerative disease, Dementia, Concussion

## Abstract

Efforts to characterize the late effects of traumatic brain injury (TBI) have been in progress for some time. In recent years much of this activity has been directed towards reporting of chronic traumatic encephalopathy (CTE) in former contact sports athletes and others exposed to repetitive head impacts. However, the association between TBI and dementia risk has long been acknowledged outside of contact sports. Further, growing experience suggests a complex of neurodegenerative pathologies in those surviving TBI, which extends beyond CTE. Nevertheless, despite extensive research, we have scant knowledge of the mechanisms underlying TBI-related neurodegeneration (TReND) and its link to dementia. In part, this is due to the limited number of human brain samples linked to robust demographic and clinical information available for research. Here we detail a National Institutes for Neurological Disease and Stroke Center Without Walls project, the COllaborative Neuropathology NEtwork Characterizing ouTcomes of TBI (CONNECT-TBI), designed to address current limitations in tissue and research access and to advance understanding of the neuropathologies of TReND. As an international, multidisciplinary collaboration CONNECT-TBI brings together multiple experts across 13 institutions. In so doing, CONNECT-TBI unites the existing, comprehensive clinical and neuropathological datasets of multiple established research brain archives in TBI, with survivals ranging minutes to many decades and spanning diverse injury exposures. These existing tissue specimens will be supplemented by prospective brain banking and contribute to a centralized route of access to human tissue for research for investigators. Importantly, each new case will be subject to consensus neuropathology review by the CONNECT-TBI Expert Pathology Group. Herein we set out the CONNECT-TBI program structure and aims and, by way of an illustrative case, the approach to consensus evaluation of new case donations.

## Introduction

There are ongoing efforts to characterize the late effects of traumatic brain injury (TBI) [18]. In recent years, attention has focused on participation in contact sports and risk of the specific neurodegenerative disease, chronic traumatic encephalopathy (CTE). However, studies have shown that CTE is not restricted solely to this population, nor is the risk of late neurodegeneration after TBI exclusively CTE [2]. Increased risk of a wide range of neurodegenerative diseases, including Alzheimer’s disease (AD) and Parkinson’s disease, has long been recognized following exposure to TBI outside of a sporting context. Indeed, an estimated 3–10% of dementia in the community is thought to be influenced by prior exposure to TBI [9, 14, 24]. Despite this we know remarkably little about the pathophysiology and pathologies of TBI-related neurodegeneration (TReND), within which CTE represents just one of several late consequences of TBI [25]. A major impediment to research progress in this field is the limited number of suitable human brain tissue specimens with linked clinical information available for research. The National Institute of Neurological Disorders and Stroke (NINDS)-supported, Center Without Walls, the COllaborative Neuropathology NEtwork Characterizing ouTcomes of TBI (CONNECT-TBI), is designed to address the need for robust, comprehensively characterized research tissue resources to support investigator-led studies in TBI.

Exposure to a TBI is acknowledged as one of the strongest environmental risk factors for early cognitive decline and dementia [5, 14, 15, 21], with a clinical phenotype typically reported similar to AD [4, 21]. However, prior studies on the etiology of dementia associated with TBI used chart reviews or clinical interviews for dementia ascertainment, which are recognized to have a low specificity [13]. No prior study of TBI-associated dementia has used pathologic confirmation of the dementia subtype, which is recognized as the gold standard [3]. Furthermore, no prior studies have used modern neurodiagnostic tools, such as neuroimaging or biomarker assays in serum or plasma, which are recognized to provide refinements over the clinical diagnosis alone [29]. Thus, although the link between TBI and dementia is acknowledged, little data exist on the precise phenotypic features and natural history of TBI-associated cognitive impairment and dementia.

Consensus neuropathological criteria for the identification of CTE neuropathologic change are derived from review of relatively few cases from a single archive. While experience suggests these criteria may be highly specific, they may not be sufficiently sensitive [16]. Furthermore, the focus on CTE, arguably, has occurred at the expense of developing a broader understanding of wider neurodegenerative outcomes and neuropathologies arising in those with a history of TBI [6, 24, 30]. For example, while the identification of CTE neuropathologic change is based on the regional pattern and distribution of hyperphosphorylated tau (pTau), multiple other proteinopathies, among other types of brain lesions, are often observed coinciding with tau abnormalities in many of the cases characterized thus far [24], including β-amyloid pathologies [26], α-synuclein pathology [1] and TDP-43 proteinopathy [17]. Furthermore, components of these pathologic changes are not exclusive to those exposed to repetitive mild TBI and have been observed after exposure to just a single moderate or severe TBI [10, 11, 27, 28].

As TReND is increasingly recognized as a major health concern [14], there is a clear and pressing need to adequately characterize the spectrum, extent and neuroanatomic distribution of the pathologies emerging in those exposed to TBI and their relationship to wider pathologies of aging and neurodegeneration. In so doing, robust operational criteria for late TBI-related neuropathologies will be defined, which will, in turn, be critical to the development of robust diagnostic, mechanistic and interventional studies. CONNECT-TBI comprises an international, multidisciplinary team of over 30 experts across 13 institutions representing unparalleled experience and resources in the investigation of the clinical and neuropathological consequences of TBI. Through this multicenter collaboration, CONNECT-TBI unites the existing, comprehensive clinical and neuropathological datasets of nine established research brain archives in TBI, several of which are unique and internationally regarded. In so doing, CONNECT-TBI provides an unrivalled, networked resource of human tissue available for research in TBI spanning diverse injury exposures and populations, with survivals ranging minutes to many decades. These existing tissue specimens will be supplemented by prospective brain banking across each site, many including research participants in ongoing, longitudinal clinical research programs studying outcomes from TBI, including Transforming Research and Clinical Knowledge in TBI (TRACK-TBI), Late Effects of TBI (LETBI), and the Concussion Assessment, Research and Education (CARE) Consortium.

## Methods

### CONNECT-TBI definitions

For the purposes of this program the following definitions are used for mild, moderate and severe TBI [22]. A mild TBI is defined as a Glasgow Coma Score (GCS) at presentation of 13–15, a loss of consciousness of less than thirty minutes, normal structural imaging and post-traumatic amnesia of less than 24 h. A moderate TBI is defined as a GCS of 9–12, loss of consciousness of more than 30 min but less than 24 h, and post-traumatic amnesia of more than 24 h but less than 7 days. A severe TBI is defined as a GCS of 3–8, loss of consciousness of more than 24 h, and post-traumatic amnesia of more than 7 days. Acute TBI is defined as survival of 6 months or less following TBI and cTBI as greater than 6 months survival from TBI.

### CONNECT-TBI program structure

The CONNECT-TBI program was formed under the umbrella of the NINDS Centers Without Walls (CWOW). This program structure is designed to bring together expertise from multiple international institutions. The CONNECT-TBI program represents a center without walls to further the field’s understanding of TBI-related neurodegeneration and associated neurocognitive decline.

**Fig. 1:**
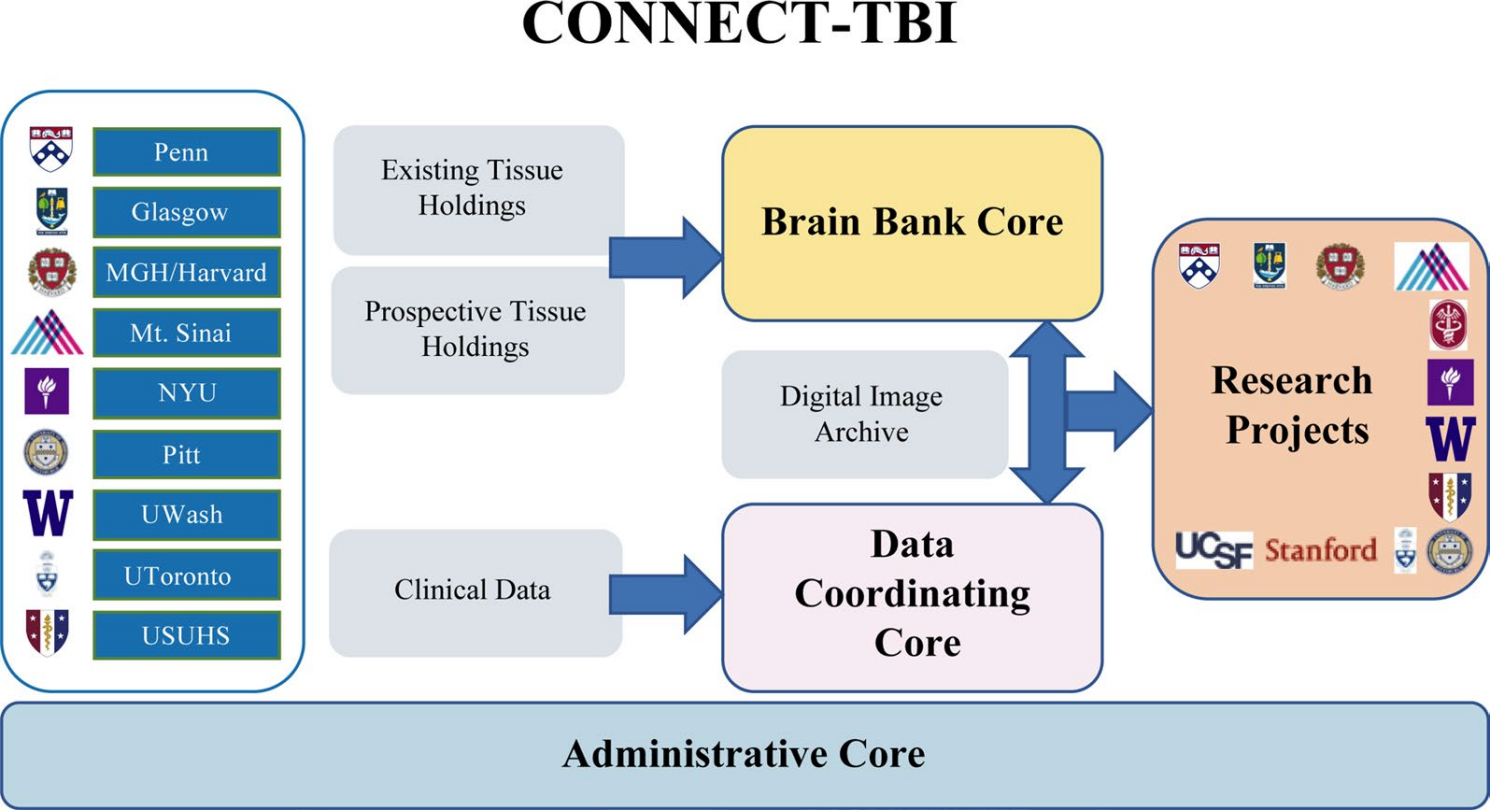
Schematic of CONNECT-TBI program

CONNECT-TBI represents a multi-site, multidisciplinary, research team working synergistically to collate a unified, central dataset of archive holdings of human tissues available for research on TBI across participating centers. In parallel, CONNECT-TBI will comprehensively characterize the neuropathological features associated with TReND and neurocognitive decline in individuals with a history of TBI and assess the contribution of key patient variables (sex, age at time of injury, survival time from injury, co-morbid medical conditions, etc.) and injury characteristics (injury severity and frequency) to these neuropathological and clinical outcomes. A critical feature of the program will be the broad sharing of clinical and neuropathological data and the development of a digital resource for distribution and sharing of fully characterized research tissue sections. The CONNECT-TBI program is a cooperative agreement with NINDS and is structured around multiple cores, namely an Administrative Core, Data Coordinating Core, and a Brain Banking Core (Fig. 1).

*The Administrative Core* coordinates the integration and management of activities within CONNECT-TBI by providing internal and external project oversight and reviewing progress against CONNECT-TBI milestones. The Administrative Core coordinates tissue archiving and access procedures for external, researcher-led enquiries, including institutional review and material transfer. To this end, the goals of the CONNECT-TBI Administrative Core include:

1. Establish multidisciplinary project oversight and review via an Internal Governance Committee and External Advisory Board.
2. Establish network governance procedures, including those to facilitate broad and enduring institutional review and material transfer agreement procedures for the CONNECT-TBI archive.
3. Create the CONNECT-TBI website which will: facilitate communication on program achievements; act as the access point for enquiries and tissue applications from external researchers; and as a central and accessible repository for all CONNECT-TBI program-generated protocols.
4. Coordinate management of data transfer from CONNECT-TBI to the Federal Interagency Traumatic Brain Injury Research (FITBIR) informatics system.
5. Oversight and guidance of CONNECT-TBI will be directed through an independent External Advisory Board and an Internal Governance Committee that will review activities against CONNECT-TBI milestones and objectives. The External Advisory Board comprises a multidisciplinary group of recognized research leaders in TBI with extensive experience in large scale, multi-center collaborative research in TBI, networked research tissue archiving in neuropathology and, researcher-led studies in TBI.

Enrollment and consent procedures and policies of each brain bank have been reviewed and approved by their respective Institutional Review Boards (IRB) and appropriate oversight committees, with central IRB approval specific to CONNECT-TBI program activities obtained from the University of Pennsylvania. The Administrative Core will ensure each collaborating brain bank center maintains their IRB approval. Material Transfer Agreements between the University of Pennsylvania and participating CONNECT-TBI institutions will allow for tissue to be freely transferred to the CONNECT-TBI central archive for staining and storage of digital scans.

**Fig. 2:**
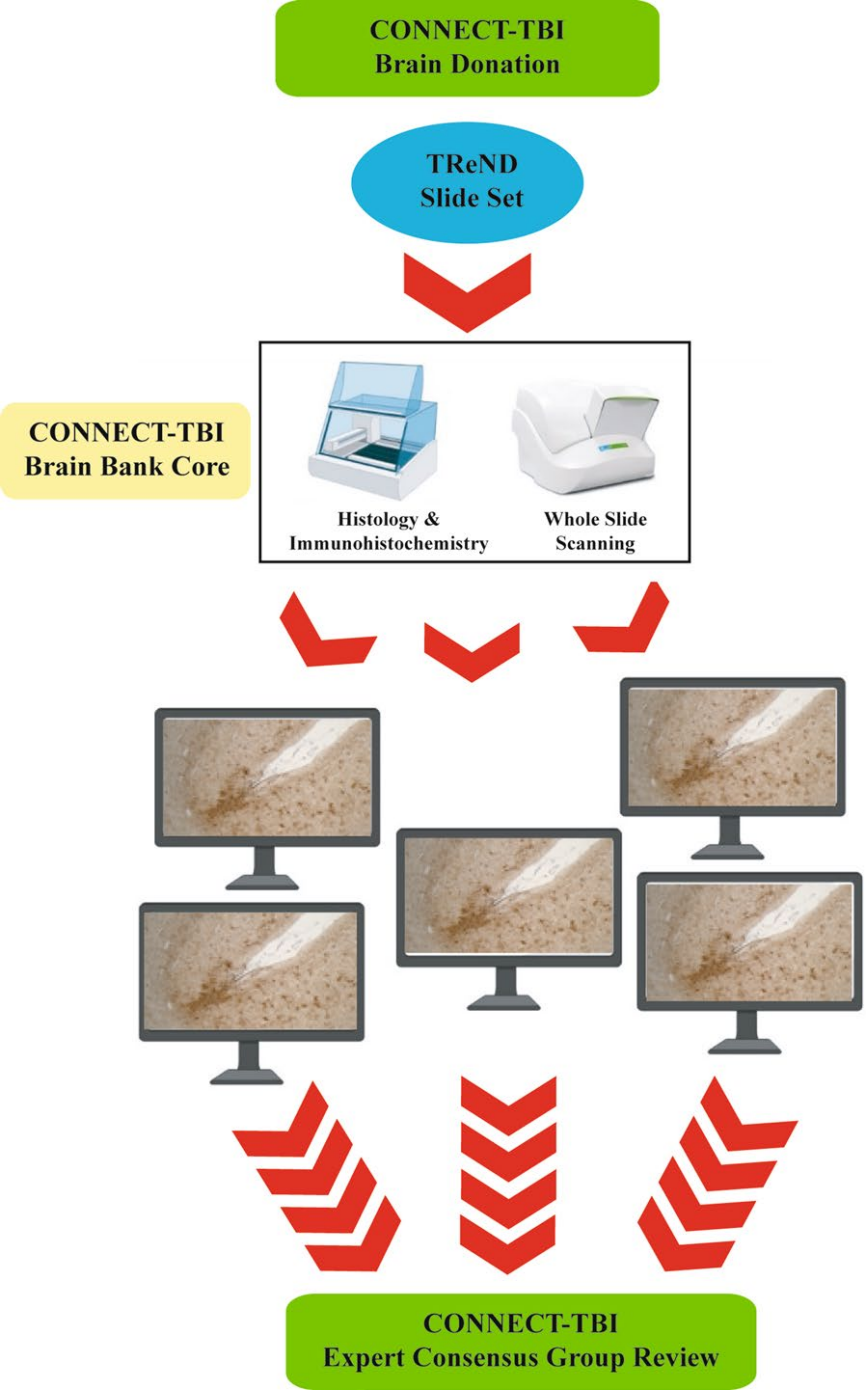
Outline of workflow, highlighting case submission, remote digital microscopy and expert consensus group review with all sites examining the same scanned section

*The Brain Bank Core* is coordinated through the University of Pennsylvania and provides a central point of access to comprehensive research resources to support global studies in outcomes from all exposure types and severities of TBI. To achieve this, the Brain Bank Core collates a centralized database of the extensive existing tissue holdings and associated clinical datasets across all CONNECT-TBI participating centers. In parallel, CONNECT-TBI facilitates coordinated research brain banking activities under harmonized protocols for the assessment and interrogation of autopsy material from patients exposed to TBI. Tissue is obtained from the following institutions: University of Pennsylvania, University of Glasgow, Harvard University, Mount Sinai, New York University, University of Pittsburgh, University of Toronto, Uniformed Services University of the Health Sciences, and University of Washington. Tissue was acquired at routine diagnostic autopsy, and approval for its use was granted by the respective Institutional Review Boards. The CONNECT-TBI Brain Bank Core serves as the coordinating and archiving center for these activities and acts as the central histologic processing site to facilitate the multi-institutional neuropathologic study of postmortem central nervous system tissues from patients exposed to TBI, including consensus group activities (Fig. 2). Eligible cases for inclusion in the CONNECT-TBI resource, are existing research archives or new case donations with history of TBI (acute or chronic) with or without history of neurodegenerative disease, patients with history of neurodegenerative disease but no known history of TBI and patients with no known history of TBI or neurodegenerative diseases as controls.

For new brain donations collected under CONNECT-TBI, each participating center generates a standardized slide set that comprises paraffin tissue sections from 16 anatomical regions, including the minimum recommended regions of the NINDS preliminary consensus protocol for the neuropathological evaluation of CTE (Table 1) [16].

**Table 1:** CONNECT-TBI standardized sampling and staining protocol

		H&E	p-tau	β-amyloid	TDP-43	α-synuclein
1	Sup frontal gyrus	X	X			
2	Mid frontal gyrus	X	X	X	X	X
3	Ant cingulate gyrus with corpus callosum	X	X	X		
4	Temporal pole	X	X		X	
5	Sup and mid temporal gyri	X	X	X		
6	Inf parietal lobule	X	X	X		
7	Hypothalamus incl mammillary body	X	X			
8	Hippocampus and entorhinal cortex	X	X	X	X	X
9	Striate cortex	X	X	X		
10	Amygdala	X	X		X	X
11	Thalamus	X	X	X		
12	Basal ganglia with nucleus basalis of Meynert	X	X	X		
13	Cerebellar cortex and dentate	X	X	X		
14	Midbrain	X	X	X		X
15	Pons	X	X			X
16	Medulla	X	X		X	X

To provide histological staining consistency, ten unstained, 8 μm formalin-fixed paraffin tissue sections from these defined brain regions will be sent to the University of Pennsylvania for standardized staining and whole slide digital imaging. Sections are stained with Hematoxylin and eosin (H&E) and immunostained for hyperphosphorylated tau (PHF-1; 1:1000, Peter Davies), β-amyloid (6F/3D; 1:75, Dako), phosphorylated TDP-43 (1D3; 1:500, Millipore), and α-synuclein (KM51; 1:200, Leica). The stained sections are then digitally scanned, with the resulting images made available to members of the CONNECT-TBI Expert Consensus Group (ECG) to assess and score pathologies in advance of quarterly diagnostic case review conference calls. The purpose of generating high quality whole-slide images in this Brain Bank Core is to allow for rapid sharing of histologic images to CONNECT-TBI investigators to support proposed Research Projects. This unique infrastructure will thus allow for all the efficiencies of local autopsy tissue procurement with centralized histologic slide staining, imaging and efficient sharing of neuropathology images.

The Expert Consensus Group will evaluate representative cases of moderate or severe TBI (n = 15); repetitive mild TBI (n = 15) or blast TBI (n = 15) and age-matched controls (n = 15). Thereafter a further randomly selected 150 cases and 50 controls representative of broad exposures and survivals in cTBI patients will be evaluated. For each case, up to 13 independent CONNECT-TBI expert neuropathologists rate brain pathologies based on recognized established or preliminary consensus criteria for the neuropathological assessment of neurodegenerative pathologies. Despite the recent focus on tau pathologies currently defining CTE, the pathologies encountered in cTBI patients often are mixed, particularly in aged patients, with multiple proteinopathies present in a single case. Thus, the prevalence of comorbid pathologies will be assessed. Specifically, each case is assessed blind to patient demographics and clinical information for the presence of:

1. Alzheimer’s disease neuropathologic changes (ADNC) [8],
2. CTE neuropathologic change (CTE-NC) [16],
3. Aging-related tau astrogliopathy (ARTAG) [12],
4. α-synuclein pathology [19],
5. TDP-43 pathology [20],
6. Cerebral amyloid angiopathy (CAA),
7. Cerebrovascular disease (CVD) [23].

In addition, comparisons will be made with other neurodegenerative disorders to determine potentially unique and shared features of TReND with wider neurodegenerative conditions and, most importantly, non-injured age-matched controls, given the importance of age as a risk factor for neurodegeneration [7].

Following review, Expert Consensus Group members return their assessments to the Brain Bank Core for collation. At this point, Expert Consensus Group members are provided anonymized clinical summaries. Agreement on consensus is based on the Royal College of Pathologists guidance for a diagnostic External Quality Assessment scheme, where above three-quarters agreement is taken as consensus. If below three-quarters agreement, the Expert Consensus Group, armed with the patient’s clinical history, reviews the pathology real-time during the consensus call using the digital scanned slide, discusses views on the pathology and agrees a consensus opinion, if possible. Where consensus cannot be achieved after this review, the discordant views are recorded.

*The Data Coordinating Core* is developing a central standardized digital neuropathological and clinical data archive, through harmonization and collation of existing neuropathological and clinical data holdings across the CONNECT-TBI network of nine leading research centers. The Data Coordinating Core integrates existing patient data resources in TBI, historically held at CONNECT-TBI network sites, into a centralized database that is shared with participating CONNECT-TBI centers and eventually with the wider research community. The Data Coordinating Core will also provide epidemiological and statistical support in analyses of data generated through associated Research Projects.

In collaboration with NINDS, key external investigators and the FITBIR program, the Data Coordinating Core will develop common data elements (CDEs) and unique data elements (UDEs) specific to TReND. These CDEs and UDEs will form the basis for all digital neuropathological data and clinical data that will be collected in all CONNNECT-TBI center projects. CDEs will be classified as being either a “core” CDE (a data element that collects essential information applicable to any study, including either those which span across all disease and therapeutic areas or those that are specific to one disease area), a “supplemental highly-recommended” CDE (a data element that is essential based on certain conditions or study types in clinical research studies), a “supplemental” CDE (a data element that is commonly collected in clinical research studies but whose relevance depends upon the study design (i.e., clinical trial, cohort study, etc.) or type of research involved), or an “exploratory” CDE (a data element that requires further validation), but may fill current gaps in the CDEs and/or substitute for an existing CDE once validation is complete.

All clinical history data for retrospective/archive cases were collected via a self- or proxy-report assessment. However, for prospective cases, participating centers will be collecting information using the Brain Injury Screening Questionnaire (BISQ) [3]; a structured and well-validated assessment which will provide a degree of standardization for the acquisition of clinical information.

The Data Coordinating Core will collate and store all data associated with the program and will provide analytical assistance in analyzing specific data. For example, the results from the neuropathology assessments of the Expert Consensus Group will be collated and outputs generated on distinction and overlap between TReND and the pathologies of wider neurodegenerative diseases and aging.

### CONNECT-TBI portal

**Fig. 3:**
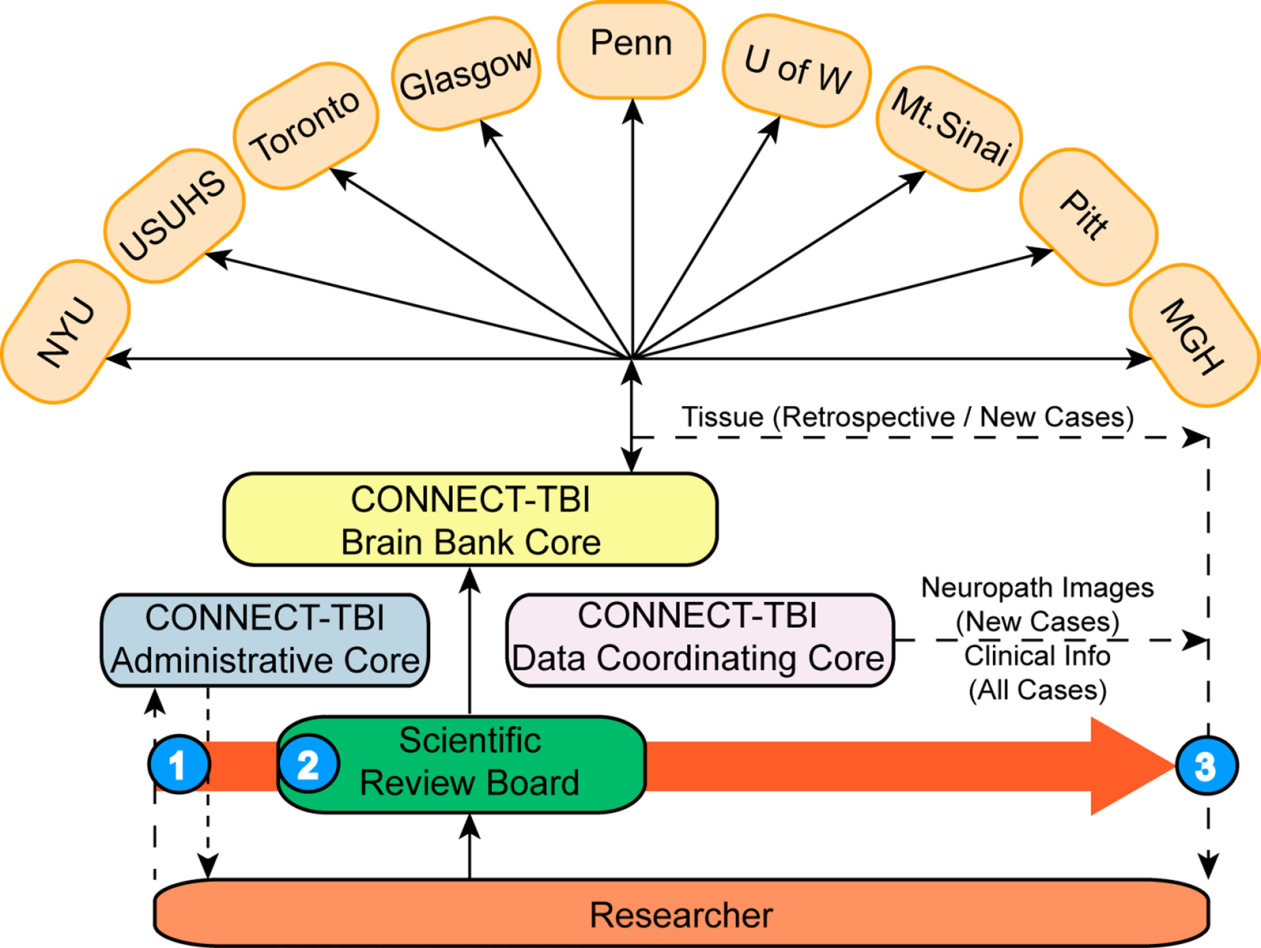
Process for research tissue sharing. (1) Researcher contacts Administrative Core in project planning phase to establish whether suitable resources within CONNECT-TBI networked archive are available to support their proposal. (2) Application submitted to Scientific Review Board for approval. (3) Following project approval by Scientific Review Board, CONNECT-TBI provides specific case numbers and institution contact details to researcher thereby initiating transfer

An overarching goal of the CONNECT-TBI program is the creation of a fully characterized research tissue resource linked to comprehensive clinical datasets to support wider, investigator-initiated enquiries in TBI. The CONNECT-TBI website facilitates access to this archive and associated pathology and clinical datasets. Requests for data or digital scans will be made through formal online applications to be approved by the CONNECT-TBI Administrative Core. Tissue requests will be directed to one of the participating CONNECT-TBI brain banks for tissue and data transfer. The CONNECT-TBI website will also host information on the overarching program aims, the investigator team and host institutions, communicate CONNECT-TBI program outputs, act as a central repository for all CONNECT-TBI program-generated ‘best practice’ protocols and procedures for archiving and assessing materials from individuals exposed to TBI, and will be the access point for tissue and image sharing (Fig. 3).

## Results

### CONNECT-TBI existing archive holdings

The current holdings across the CONNECT-TBI program comprise research tissue samples from almost 2000 acute TBI (aTBI) and over 900 chronic TBI (cTBI) cases. Regarding cTBI cases, these comprise 559 civilian, 143 sport (e.g. football, soccer, hockey, rugby, boxing) and 268 former military personnel (Table 2).

**Table 2:** Current tissue holdings of acute and chronic TBI across CONNECT-TBI centers

	Civilian		Sport		Military		Total	
	aTBI	cTBI	aTBI	cTBI	aTBI	cTBI	aTBI	cTBI
University of Glasgow	1654	139	5	25	0	1	1659	165
University of Pennsylvania	3	90	0	26	0	18	3	134
Massachusetts General	16	2	0	0	0	0	16	2
Mount Sinai	0	22	0	8	0	6	0	36
New York University	295	77	0	0	0	7	295	84
University of Pittsburgh	3	55	0	13	0	1	3	69
University of Toronto	0	6	2	50	0	0	2	56
University of Washington	8	159	0	19	0	46	8	224
USUHS	7	9	0	2	5	189	12	200
Total	1986	559	7	143	5	268	1998	970

*aTBI* acute survival from traumatic brain injury, *cTBI* chronic survival from traumatic brain injury, *USUHS* Uniformed Services University of the Health Sciences

From each CONNECT-TBI center, standardized CDEs and UDEs on each case are being collated to the central CONNECT-TBI archive dataset to include, where available: patient demographics (including age, sex, ethnicity); TBI injury details including mechanism, severity, medical interventions and survival interval; clinical recovery (including estimates of extended Glasgow Outcome Scale, cognitive function, post-traumatic epilepsy); documented known medical comorbidities; tissue samples archived (including anatomical locations, whether fixed or frozen tissue available); and results of neuropathological assessments (including assessments of neurodegenerative disease pathologies). Review of the CONNECT-TBI centers with research tissue banks in neurodegeneration, identifies tissue samples from over 2000 donors with AD, over 850 donors with Parkinson’s/Lewy body disease and over 3500 with other neurodegenerative diseases, such as frontotemporal lobar degeneration, in addition to material from normal controls with no known history of TBI or neurodegenerative disease (Table 3).

**Table 3:** Current tissue holdings in neurodegenerative disease available within CONNECT-TBI centers

	Alzheimer's	Parkinson's/Lewy Body	Other	Total
University of Pennsylvania	627	386	758	1771
University of Pittsburgh	808	81	794	1683
University of Washington	858	407	2096	3361
Total CONNECT-TBI NDD	2293	874	3645	6815

*NDD* neurodegenerative disease

### CONNECT-TBI new case consensus review and archiving

All CONNECT-TBI centers contribute material from new research brain donations for Expert Consensus Group diagnostic review and archiving. In year 1 of CONNECT-TBI, a total of 141 cases have been accrued across centers: 36 from individuals with a history of TBI and/or exposure to repetitive head impacts, the remainder as non-TBI associated neurodegenerative disease (n = 100) or controls with no history of TBI or neurodegenerative disease (n = 5). Demographic information and neuropathological diagnoses for these cases are shown in Table 4.

**Table 4:** Demographic information and neuropathological diagnoses for Year 1 CONNECT-TBI new case donations

	History of TBI	No history of TBI
Number of cases	36 (26%)	105 (74%)
Male	27 (75%)	58 (55%)
Mean age (range)	65 (20–93)	75 (31–100)

	n (%)	n (%)
*TBI exposure*		
Sport	14 (39)	NA
Military	3 (8)	NA
Civilian	33 (92)	NA
Multiple	7 (19)	NA
*Neuropathology*		
CTE-NC	8 (22)	0 (0)
ADNC	20 (56)	71 (68)
LBD	8 (22)	42 (40)
ARTAG	12 (33)	48 (46)
FTLD	2 (6)	9 (9)
CVD	3 (8)	32 (31)

*ADNC* Alzheimer’s disease neuropathologic changes, *ARTAG* aging-related tau astrogliopathy, *CTE-NC* chronic traumatic encephalopathy neuropathologic change, *CVD* cerebrovascular disease, *FTLD* frontotemporal lobar degeneration, *LBD* Lewy body disease, *NA* not applicable, *TBI* traumatic brain injury

### Index case

#### Clinical history

A subject who died in his 70s first reported notable symptoms in his early 60s. At that time, he noticed mild incoordination, with associated impaired visuospatial awareness, leading to his frequently bumping into stationary objects. He had also developed a shuffling gait, with occasional instances where he would catch his foot on a floor surface and trip, without falling. In the following years he reported increasingly vivid, distressing dreams and visual hallucinations, which he eventually lost insight to. These evolving symptoms were associated with mood change, which his family described as a somewhat flat affect. Approximately 6 years after symptom onset, he developed difficulties with speech, with limiting of vocabulary and hesitancy on production. He was initially examined by a primary care physician who noted no apparent deficit in routine clinical or memory screening tests, but nevertheless arranged for review by secondary care mental health services. At this review, formal cognitive assessment with Addenbrooke’s Cognitive Examination (ACE) noted a score of 81/100, and an initial diagnosis of mild cognitive impairment (amnestic type) was suggested; later revised to Alzheimer’s dementia when functional abilities were lost. Treatment with psychosocial interventions and acetylcholinesterase inhibitor medication was commenced. In the years that followed, there was a steady decline in his condition requiring increasing assistance with self-care, such that 3 years after this initial review and diagnosis he was transitioned to residential care for ongoing support, where he remained until his death.

The subject had a history of mild anxiety and depressive symptoms noted from his 50s, which was managed by his primary physician and did not require psychotropic medication or inpatient services. There was no documented history of suicidal ideation. Other than prior knee surgeries, the patient is described as having been in excellent health and had no history of alcohol or recreational drug dependency. There is no notable family history of neurodegenerative disease or of mental disorder.

The patient was a former soccer player (amateur and professional), playing as a defender and retiring in his mid-30s. During his sporting career, he is known to have sustained at least two mild traumatic brain injuries with documented brief loss of consciousness with nonspecific symptomology in the following 2-week period. No other history of TBI was noted. The patient did not participate in combat sports and had no history of military service.

#### Post-mortem findings

**Fig. 4:**
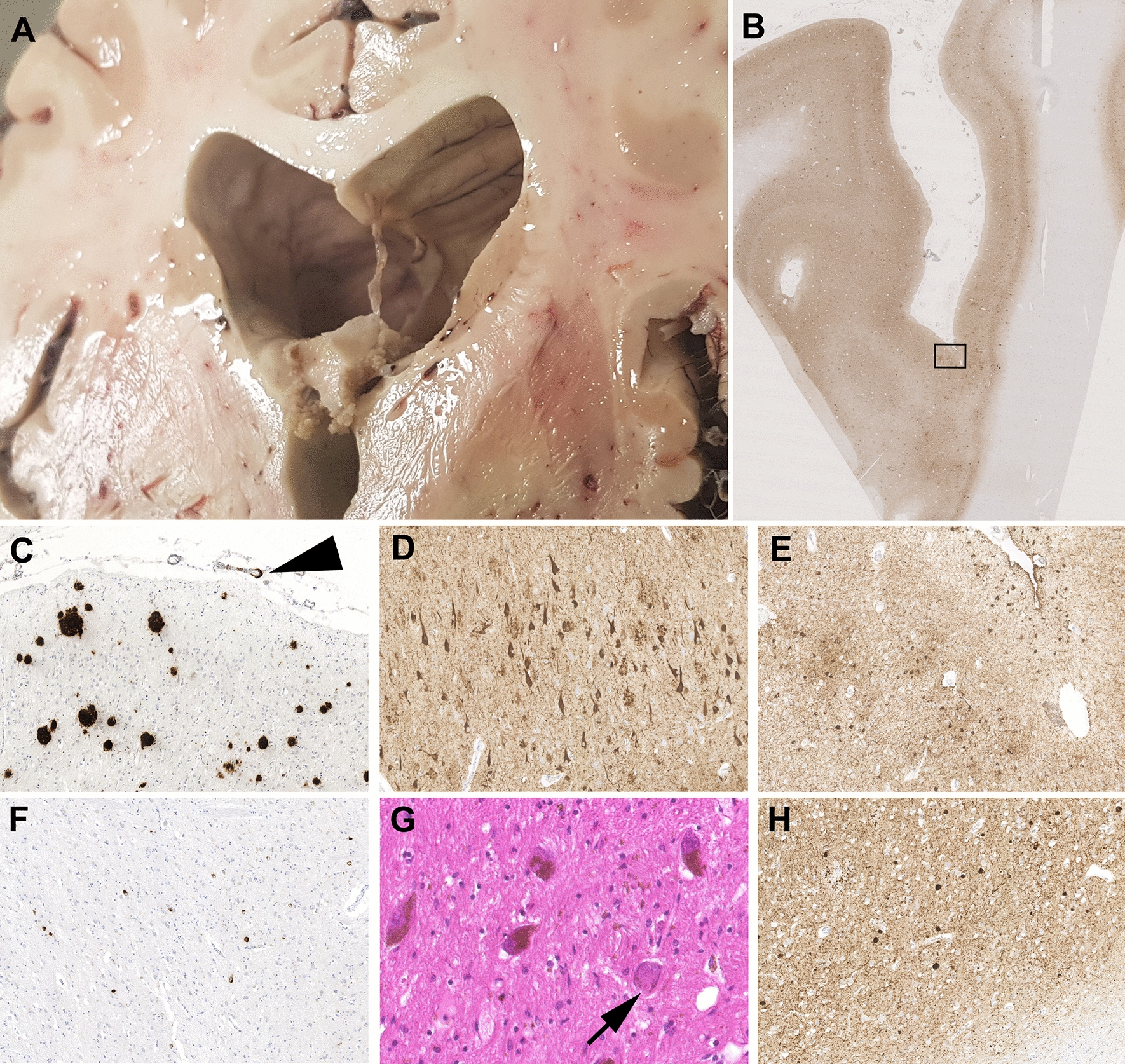
Representative images of pathology of illustrative case. Fenestration of the septum pellucidum (**a**). Cortical pTau in neurons and glia consistent with neurofibrillary tangles of Alzheimer’s disease neuropathologic change and astrocytic pathology of aging-related tau astrogliopathy (**b**, **e**; PHF-1), with abundant neurofibrillary tangles also present in hippocampal sector CA1 (**d**; PHF-1). In addition, frequent neuritic amyloid plaques and cerebral amyloid (arrowhead) were present (**c**; 6f3d). Elsewhere, numerous cytoplasmic inclusions immunoreactive for phospho-TDP-43 were identified in the dentate granule cell layer (**f**; 1D3). Within the substantia nigra numerous classical Lewy bodies were present (**G**; arrow), with frequent cortical Lewy bodies revealed in staining for alpha-synuclein (**h**; KM51)

At autopsy, the intact, formalin-fixed brain weighed 1400 g, with the hindbrain weight 195 g. The cerebral hemispheres were symmetrical with a normal lobulation and gyral architecture. Mild atrophy of the gyri over the frontal and temporal poles was noted, associated with widening of the sulci and gelatinous thickening of the overlying meninges. Otherwise the external appearances were unremarkable. On sectioning the cerebral hemispheres in the coronal plane, there was noted fenestration of the septum pellucidum, particularly towards the posterior extent where it was virtually absent (Fig. 4). Elsewhere there was cortical atrophy with thinning of the frontal and temporal gyri, bilateral hippocampal atrophy and ventriculomegaly. The cerebellum appeared unremarkable externally and on sectioning, with no notable abnormalities in the brainstem; the substantia nigra pigmentation appearing within normal limits for the patient’s age. There was no macroscopic evidence of recent or previous TBI.

Systemic examination revealed macroscopic evidence of abundant yellow fluid emerging from the cut surfaces of the airways of the lower and posterior segments of the lungs bilaterally. Histological sections of the lungs confirmed features of an established bronchopneumonia in association with scattered clusters of rounded bacterial organisms. Otherwise, the systemic examination was essentially unremarkable.

#### Neuropathology expert consensus evaluation

Multiple brain tissue samples were processed consistent with the CONNECT-TBI new case evaluation protocol, with the resultant stained sections then scanned and distributed to the Expert Consensus Group for review and assessment of TReND pathologies (Fig. 4). Ten of 13 members of the Expert Consensus Group provided independent evaluations of the pathologies in this case, with consensus achieved for multiple evaluations at this first review (Table 5).

**Table 5:** Independent neuropathological assessments of illustrative case

	ADNC				CTE-NC		α-synuclein		TDP-43	ARTAG	CAA	CVD
	A	B	C	ADNC	Absent/present	Stage	Absent/present	Stage	Absent/present	Absent/present	Absent/present	Severity
Pathologist 01	3	3	3	High	Absent	NA	Present	Diffuse neocortical	Present	Present	Present	Moderate
Pathologist 02	3	2	3	Inter	Absent	NA	Present	Cortex and brainstem	Present	Absent	Absent	None
Pathologist 03	2	2	3	Inter	Absent	NA	Present	Neocortical	Absent	Present	Absent	None
Pathologist 04	3	3	3	High	Absent	NA	Present	Amygdala/cortical/brainstem	Present	Absent	Absent	None
Pathologist 05	2	3	3	Inter	Absent	NA	Present	Neocortical	Present	Present	Present	Mild
Pathologist 06	2	3	2	Inter	Absent	NA	Present	Neocortical	Present	Present	Absent	None
Pathologist 07	3	3	3	High	Absent	NA	Present	Neocortical	Present	Present	Present	Mild
Pathologist 08	3	2	3	Inter	?	?	Present	Neocortical	Present	Absent	Absent	Mild
Pathologist 09	3	3	3	High	Absent	NA	Present	Neocortical	Present	Present	Present	NA
Pathologist 10	3	3	3	High	Absent	NA	Present	Neocortical	Present	Present	Present	Mild
Consensus opinion	2*	3	3	Inter	Absent	NA	Present	Neocortical	Present	Present	Present	None/mild

*ADNC* Alzheimer’s disease neuropathologic changes, *ARTAG* aging-related tau astrogliopathy, *CAA* cerebral amyloid angiopathy, *CTE-NC* chronic traumatic encephalopathy neuropathologic change, *CVD* cerebrovascular disease, *Inter* intermediate, *NA* not applicable

The remaining pathologies for which no immediate consensus was achieved were discussed during a videoconference call between the Expert Consensus Group with access to the scanned slides (13/16 ECG members were present). This resulted in consensus being achieved for all pathologies under evaluation, with a final integrated diagnosis of neurodegenerative disease recorded as dementia with Lewy bodies, with noted comorbid pathologies as: intermediate ADNC; TDP-43 proteinopathy; ARTAG; CAA; and low CVD pathology (Fig. 4).

## Discussion

CONNECT-TBI has established a network of internationally recognized brain banks and academic institutions creating a comprehensive tissue and data resource in human TBI to support studies by the wider research community. Among the available resources is case material from patients across all injury survival intervals, and from across the broad spectrum of injury subtypes, including civilian, military and sports. Leveraging these resources, CONNECT-TBI has begun to characterize the extent, distribution and range of neuropathologies that result following exposure to TBI, specifically the pathologies associated with TReND.

The CONNECT-TBI network will unify characterized tissue resources and comprehensive clinical data sets which will advance research into the neuropathological features of TReND, healthy ageing, and neurodegenerative diseases more widely. However, it is recognized that an unavoidable limitation of the network design is a reliance on self- or proxy-report assessment in clinical histories, which may be incomplete for archive cases. Investigating associations of neuropathological and clinical outcomes following TBI with a range of patient variables might therefore be challenging. However, for prospective cases, participating centers will be asked to collect information using the Brain Injury Screening Questionnaire (BISQ); a structured and well-validated assessment which will provide a degree of standardization for the acquisition of clinical information.

With the increasingly global nature of research, sustaining major international collaborations can present challenges, particularly those that require detailed examination of human tissue. CONNECT-TBI has shown that a centralized archive of digitally scanned stained tissue sections can be distributed, examined and assessed efficiently using established neuropathological criteria and in a timely manner. Supplemented with virtual group discussions that include live slide examinations, there is opportunity for continued case review and consensus diagnostic evaluation of the spectrum of TReND.

Although animal models can provide valuable information, particularly with respect to examining specified time points and investigating potential mechanisms of injury, there are considerable recognized limitations of pre-clinical studies in informing on human TBI. To date, in excess of 30 clinical trials of candidate therapies for TBI have failed, despite early promise from pre-clinical studies. The multidisciplinary expertise contributing to the CONNECT-TBI program provides opportunity for rigorous, unbiased evaluation of both clinical and neuropathological facets of TReND that can be used to inform mechanistic studies and therapeutic strategies. Current consensus criteria for the neuropathological assessment of CTE are based on a limited number of cases. The number of cases required to characterize TReND comprehensively exceeds the capability of individual biorepositories. Availability of human tissues for research purposes is often impeded by the demands on expertise and resources required to establish suitable tissue banking facilities. CONNECT-TBI aims to fill this void by providing a central point of access to multiple centers for both physical and virtual tissue.

## Conclusions

A central brain tissue repository with access to clinical data in large archives of fully characterized TBI and neurodegenerative disease cases, with appropriate age-matched non-injured controls and associated clinical data, will be a priceless resource to the greater TBI research community. This resource will be utilized to generate a consensus in the operational criteria for the diagnosis of TReND across all range and subtypes and to evaluate the extent and distribution of all neuropathologies resulting from TBI exposure. Furthermore, the center will seek to contrast the phenotypes of TReND with that of wider neurodegenerative disease and with aging processes. In all, the CONNECT-TBI collaboration will represent a broad, comprehensive exploration of the intricate neuropathological changes following TBI.

## Data Availability

The datasets used during the current study is available from the corresponding authors on reasonable request.

## Abbreviations

AD: Alzheimer’s disease
ADNC: Alzheimer’s disease neuropathologic changes
ARTAG: aging-related tau astrogliopathy
aTBI: acute traumatic brain injury
CAA: cerebral amyloid angiopathy
CDE: common data elements
CONNECT-TBI: COllaborative Neuropathology NEtwork Characterizing ouTcomes of TBI
cTBI: chronic traumatic brain injury
CTE: chronic traumatic encephalopathy
CTE-NC: CTE neuropathologic change
CVD: cerebrovascular disease
CWOW: center without walls
ECG: expert consensus group
FITBIR: federal interagency traumatic brain injury research
H&E: hematoxylin and eosin
IRB: Institutional Review Board
pTau: phosphorylated tau
TBI: traumatic brain injury
TReND: traumatic brain injury related neurodegeneration
pTau: phosphorylated tau
UDE: unique data elements
